# An Adult Brain Atlas Reveals Broad Neuroanatomical Changes in Independently Evolved Populations of Mexican Cavefish

**DOI:** 10.3389/fnana.2019.00088

**Published:** 2019-10-04

**Authors:** Cody Loomis, Robert Peuß, James B. Jaggard, Yongfu Wang, Sean A. McKinney, Stephan C. Raftopoulos, Austin Raftopoulos, Daniel Whu, Matthew Green, Suzanne E. McGaugh, Nicolas Rohner, Alex C. Keene, Erik R. Duboue

**Affiliations:** ^1^Department of Biology, Charles E. Schmidt College of Science, Florida Atlantic University, Jupiter, FL, United States; ^2^Jupiter Life Science Initiative, Florida Atlantic University, Jupiter, FL, United States; ^3^Stowers Institute for Medical Research, Kansas City, MO, United States; ^4^Harriet L. Wilkes Honors College, Florida Atlantic University, Jupiter, FL, United States; ^5^Department of Ecology, University of Minnesota, St. Paul, MN, United States; ^6^Department of Molecular and Integrative Physiology, KU Medical Center, Kansas City, KS, United States

**Keywords:** *A. mexicanus*, hypothalamus, brain evolution, brain atlas, sleep, stress, feeding

## Abstract

A shift in environmental conditions impacts the evolution of complex developmental and behavioral traits. The Mexican cavefish, *Astyanax mexicanus*, is a powerful model for examining the evolution of development, physiology, and behavior because multiple cavefish populations can be compared to an extant, ancestral-like surface population of the same species. Many behaviors have diverged in cave populations of *A. mexicanus*, and previous studies have shown that cavefish have a loss of sleep, reduced stress, an absence of social behaviors, and hyperphagia. Despite these findings, surprisingly little is known about the changes in neuroanatomy that underlie these behavioral phenotypes. Here, we use serial sectioning to generate brain atlases of surface fish and three independent cavefish populations. Volumetric reconstruction of serial-sectioned brains confirms convergent evolution on reduced optic tectum volume in all cavefish populations tested. In addition, we quantified volumes of specific neuroanatomical loci within several brain regions that have previously been implicated in behavioral regulation, including the hypothalamus, thalamus, and habenula. These analyses reveal an enlargement of the hypothalamus in all cavefish populations relative to surface fish, as well as subnuclei-specific differences within the thalamus and prethalamus. Taken together, these analyses support the notion that changes in environmental conditions are accompanied by neuroanatomical changes in brain structures associated with behavior. This atlas provides a resource for comparative neuroanatomy of additional brain regions and the opportunity to associate brain anatomy with evolved changes in behavior.

## Introduction

Shifts in environmental conditions drive evolutionary changes in development, morphology, and behavior (Peichel et al., 2001; Shapiro et al., 2004; Jeffery, 2009). While the genetic basis of many behaviors has been studied extensively, much less is known about how changes in brain anatomy accompany behavioral evolution. Interspecies comparative approaches are often used to associate anatomical or neural circuit changes with evolved behavioral differences (Shapiro et al., 1991; Jarvis, 2004; Schenker et al., 2008). However, these studies often focus on individual brain regions of interest and interpretations may be limited by the indirect nature of comparing different species. The generation of detailed brain atlases of distinct populations of the same species with divergent behavioral traits has potential to provide insight into the relationship between neuroanatomical evolution and behavior.

The Mexican cavefish, *Astyanax mexicanus*, provides the unique opportunity to investigate the relationship between brain anatomy and behavioral evolution in a single species (Yoshizawa et al., 2010; Elipot et al., 2013; Alié et al., 2018; Jaggard et al., 2018; Lloyd et al., 2018). These fish exist as an eyed, pigmented population that inhabits the rivers and streams of northeast Mexico and southern Texas, and at least 29 independent populations of largely blind and depigmented fish that inhabit the caves of northeast Mexico’s Sierra de El Abra and Sierra de Guatemala regions (Mitchell et al., 1977). Both surface and cave populations are interfertile, which allows for direct comparisons of populations from the same species with different and well-described habitats and evolutionary history (Şadoğlu, 1957; Wilkens, 1971). Comparisons between surface fish and cavefish populations reveal evolved differences in diverse behavioral traits ranging from social behavior to sleep, and the emergence of these behaviors in multiple cavefish populations has established *A. mexicanus* as a model for convergent evolution (Duboué et al., 2011; Elipot et al., 2013; Kowalko et al., 2013a, b; Chin et al., 2018).

A number of neuroanatomical differences have been identified between surface fish and cavefish, including a reduction in brain regions associated with visual processing in cavefish, and an expansion of the hypothalamus, which is associated myriad behaviors including social interaction, aggressive, and sleep (Soares et al., 2004; Menuet et al., 2007; Alié et al., 2018; Jaggard et al., 2018). Nevertheless, *A. mexicanus* lacks a detailed brain atlas, and little is known about the extent of neuro anatomical changes between individual populations of cavefish. Further, the resources for a whole-brain anatomical comparison between adult cave populations have not been developed, and it remains unclear if distinct or shared changes in brain anatomy underlie the behavioral differences observed between independently evolved cavefish populations.

Here, we used serial sectioning of Nissl-stained brains, followed by volumetric reconstruction to generate brain atlases for surface fish and three different populations of cavefish. Our analysis focuses on hypothalamic, thalamic and prethalamic, and habenular regions, which have previously been associated with behaviors known to diverge between surface fish and cavefish including responses to stress, social behavior, sleep regulation, feeding, and sensory processing (Duboué et al., 2011; Elipot et al., 2013; Kowalko et al., 2013a, b; Chin et al., 2018; Jaggard et al., 2018). Our findings reveal an expansion of thalamic and other regions in cavefish, accompanied by a reduction in regions associated with visual processing. Strikingly, some hypothalamic nuclei are enlarged in cavefish, while other hypothalamic regions remain unchanged. Together, these findings provided a detailed anatomical reference for *A. mexicanus* and provide insight into the anatomical plasticity that accompanies the evolution of multiple behaviors.

## Results

### Volumetric Reconstruction of Serial Sectioned Adult Brains

**FIGURE 1:**
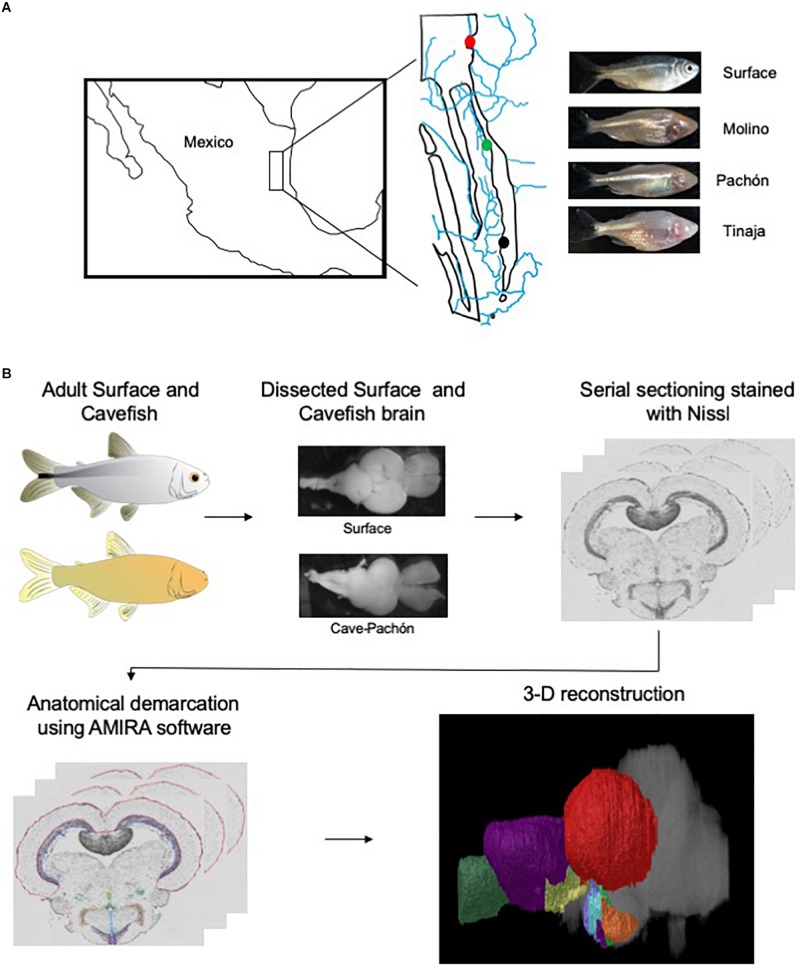
Overview of experimental design. **(A)** Map of Mexico with location of Molino (red), Pachón (green) and Tinaja (black) caves. **(B)** Flow chart diagraming experimental design and procedure.

To generate an adult brain atlas, we serially sectioned brains of adult *A. mexicanus* from surface fish and three independent populations of cavefish: Pachón, Molino and Tinaja (Figure 1A). The Pachón and Tinaja populations are “old lineage” and are closely related, while fish from the Molino population represent a “new lineage” (Bradic et al., 2012). All cave populations are thought to have evolved independent of one another (Ornelas-García et al., 2008; Bradic et al., 2013; Herman et al., 2018). Surface fish used in this experiment are derived from a lineage that is more closely related to the Molino cave fish population than to Tinaja and Pachón (Herman et al., 2018). Brains were dissected from adult animals, sectioned serially at 8 μm thickness, stained with cresyl violet dye (Nissl), and imaged, resulting in 424–728 sections per brain (Table 1). We then aligned all brain slices using image registration techniques so that they aligned with one another, and imported the data into AMIRA 3D rendering software, where serial-sections were volumetrically reconstructed to generate a three-dimensional brain (Figure 1B and Supplementary Movies S1–S4).

**TABLE 1:** Fish metrics.

**Population**	**Individual**	**Sex**	**Body length (mm)**	**Weight (g)**	**Volume of entire brain (mm^3) (not standardized)**	**Volume of entire brain/length of fish**	**Slices per brain**
Surface	1	Male	44.26	1.73	6.029	0.136	424
Surface	2	Female	46.15	2.01	6.93	0.15	533
Pachon	1	Female	50.21	3.04	6.49	0.129	650
Pachon	2	Male	48	2.53	9.05	0.189	614
Tinaja	1	Male	45.11	2.22	4.92	0.109	451
Tinaja	2	Female	48.17	2.75	6.003	0.125	667
Tinaja	3	Female	47.86	2.64	15.86	0.331	728
Molino	1	Female	45.64	2.09	6.712	0.147	591
Molino	2	Male	39.8	1.34	4.97	0.125	537

Selected neuroanatomical regions in each brain were identified by comparing to an adult zebrafish brain atlas (Wulliman et al., 1996), and a previously annotated brain of cavefish from the Micos cave (Natke, 1999), a hybrid cave population (a cave with fish with that have both surface and cave-like traits) of the new lineage (Bradic et al., 2013). After locating individual neuroanatomical regions, we defined each brain nucleus by demarcating the boundaries of the region throughout serial sections using AMIRA (see section “Materials and Methods;” Figure 1B). We then quantified the volume of each region. The volume of each quantified region was normalized to the length of the fish, measured from the nose to the caudal tail, providing a measurement of relative volumetric enlargement or reduction in size between *A. mexicanus* populations (Table 2).

**TABLE 2:** Raw Data of neuroanatomical loci.

**Population**	**Individual**	**Sex**	**Optic Tectum (mm^3)**	**Paraventricular Gray Zone of Optic Tectum (mm^3)**	**Telencephalon (mm^3)**	**Thalamus (mm^3)**	**Posterior Thalamic Nucleus (mm^3)**	**Anterior Thalamic Nucleus (mm^3)**	**Lateral Prethalamus (mm^3)**	**Ventromedial Thalamic Nucleus Medial Prethalmus (mm^3)**	**Intermediate Thalamic Nucleus (mm^3)**	**Central Posterior Thalamic Nucleus (mm^3)**	**Habenula (mm^3)**	**Dorsal Habenular Nucleus (mm^3)**	**Ventral Habenular Nucleus (mm^3)**	**Habenular Commissure (mm^3)**	**Hypothalamus (mm^3)**	**Suprachiasmatic Nucleus (mm^3)**	**Lateral Hypothalamus (mm^3)**	**Dorsal Zone of the Periventricular Hypothalamus (mm^3)**	**Caudal Zone of the Periventricular Hypothalamus (mm^3)**	**Ventral Zone of the Periventricular Hypothalamus (mm^3)**	**Paraventricular Organ (mm^3)**	**Preoptic Nucleus (mm^3)**	**Anterior Tuberculum (mm^3)**	**Posterior Tuberculum (mm^3)**
Surface	1	Male	1.09	0.2235	0.7053	0.023	0.00159	0.000649	0.00185	0.00169	0.000368	0.0134	0.006	0.00224	0.00344	0.00032	0.1144	0.0003	0.00608	0.02469	0.00816	0.00752	0.002099	0.0213	0.03637	0.00785
Surface	2	Female	1.1998	0.20737	0.93731	0.0152	0.001175	0.000657	0.006416	0.00096	0.000421	0.003885	0.0082	0.004133	0.00391	0.00019	0.1299	0.004969	0.00395	0.03302	0.01525	0.00831	0.00112	0.02674	0.03096	0.00556
Pachon	1	Female	0.86	0.2325	2.955	0.0285	0.003185	0.00212	0.0044	0.00235	0.000865	0.0112	0.0175	0.01032	0.00686	0.000361	0.3882	0.00556	0.0205	0.08499	0.05893	0.03087	0.00449	0.07312	0.08477	0.025
Pachon	2	Male	0.7175	0.193888	1.5	0.0279	0.002765	0.004035	0.00572	0.00195	0.0006355	0.009449	0.0117	0.00649	0.00498	0.0002555	N/A	N/A	0.0125	0.0573	0.0296	0.0175	N/A	N/A	N/A	N/A
Tinaja	1	Male	0.2333	0.09418	0.89392	0.0141	0.00222	0.0020951	0.003933	0.002464	0.0007315	0.00147668	0.0091	0.002823	0.005919	0.000313	0.1128	0.003003	0.005463	0.024614	0.01482	0.006912	0.001924	0.025177	0.025167	0.0057235
Tinaja	2	Female	0.364	0.1352	1.166	0.0177	0.00245	0.000942	0.00205	0.00267	0.000676	0.005207	0.0115	0.004375	0.006585	0.000554	0.1936	0.0045	0.012711	0.04035	0.01422	0.01869	0.003944	0.0331	0.04733	0.01874
Tinaja	3	Female	N/A	N/A	N/A	N/A	N/A	N/A	N/A	N/A	N/A	N/A	0.0157	0.00784	0.00769	0.000125	0.411	0.0115	0.0199	0.125	0.0506	0.026	0.00634	0.0637	0.082	0.026
Molino	1	Female	0.5258	0.127	1.172	0.0196	0.00223	0.003958	0.00453	0.001957	0.0004112	0.00465	0.01356	0.004923	0.00862	0.00002139	0.3993	0.004412	0.00838	0.0469	0.01666	0.014664546	0.004413	0.02534	0.0412	0.0092
Molino	2	Male	0.4469	0.14922	2.8856	0.0263	0.002036	0.00547	0.00707355	0.00274	0.0009688	0.00585	0.0359	0.0165	0.0178	0.001565	0.4324	0.00848	0.0159	0.11774	0.0649	0.02386	0.005744	0.0809	0.0872	0.02745

### Regression of Optic Tectum Volume in Cavefish Populations

**FIGURE 2:**
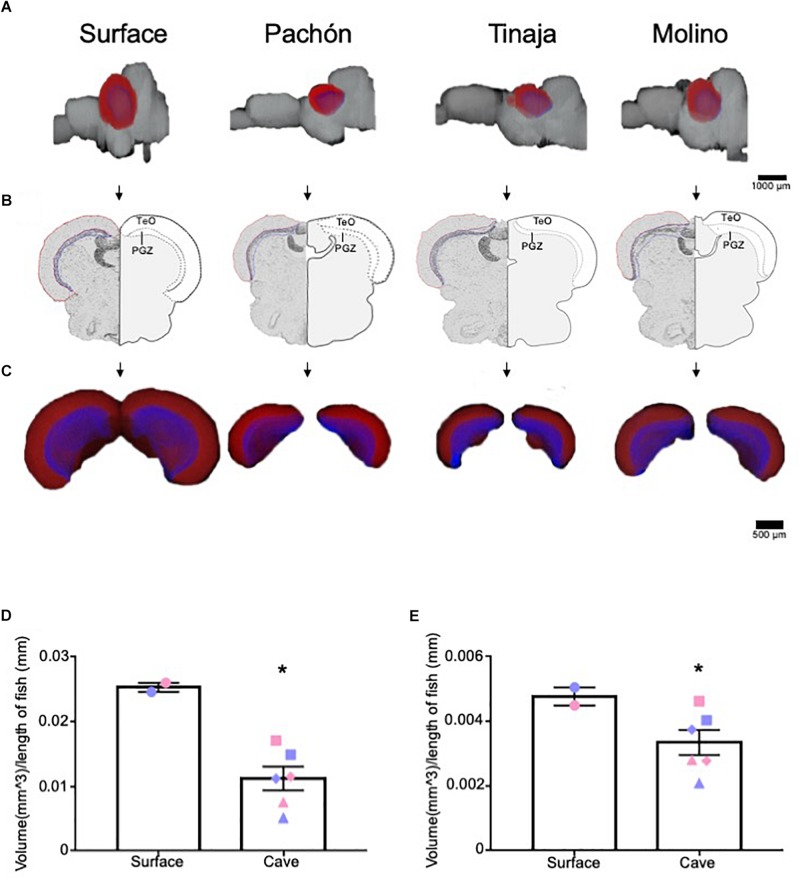
Three-dimensional reconstruction reveals regression of the optic tectum. **(A)** 3-D reconstructions of Surface, Pachón, Tinaja and Molino with optic tectum (TeO; red) and periventricular gray zone of the optic tectum (PGZ; blue) displayed. **(B)** Images of sections that were Nissl stained (left), and a cartoon of the demarcated TeO and PGZ shown (right). **(C)** 3-D reconstruction of the TeO and PGZ. Displayed from an anterior view. **(D)** Quantification of the volume of optic tectum, normalized to the length of the animal for surface fish and for the three cave populations. Optic tectum of cavefish was significantly smaller than those of surface fish conspecifics (surface fish 0.025 ± 0.0007, cavefish 0.011 ± 0.0018, *t*-test *t* = 4.224, df = 6, *p* < 0.05). Quantification of the volume of PGZ. The PGZ of most cavefish was smaller than those of surface animals (surface fish 0.005 ± 0.0003, cavefish 0.003 ± 0.0004, *t*-test *t* = 1.987, df = 6, *p* < 0.05). Graphs in **D** and **E** are the mean ± standard error of the mean. Asterisk represent significance below *p* = 0.05. Blue shapes on bar graphs denote males, whereas light red shapes denote females. Squares on graphs represent Pachón, triangles represent Tinaja and diamonds represent Molino.

The optic tectum of *A. mexicanus* has been well studied, and the size, as well as afferent and efferent projections from the optic tectum have been established (Sligar and Voneida, 1976; Voneida and Sligar, 1976; Soares et al., 2004). As proof of principle, we first quantified the volume of the optic tectum (Figures 2A–C, red) and it’s corresponding periventricular gray zone (PGZ) (Figures 2A–C, blue), which have been reported as reduced in Pachón cavefish (Soares et al., 2004; Moran et al., 2015). The optic tectum in adult teleosts is a laminated structure. We measured the PGZ, which contains most cell bodies, as well the optic tectum minus the PGZ (TeO), which contains most fibers and synaptic connections. In agreement with previous findings, whole-brain reconstructions revealed a nearly two-fold reduction in the tectum size of Pachón, Molino and Tinaja cavefish compared to surface fish (Figures 2A–C). To increase power to detect statistical significance, we combined the total volume of the optic tectum of all cave populations and compared them to surface fish. This comparison revealed significant differences in volume between surface and cave morphs (Figure 2D). Quantification of total volumes between surface and the three cave populations revealed a substantial reduction in total volume (Pachón = 36.7% decrease in volume compared to surface fish, Tinaja = 76.2% decrease in volume compared to surface fish, Molino = 56.5% decrease in volume compared to surface fish). In addition to the optic tectum, the volume of the PGZ appeared qualitatively reduced across all three cave populations (Figure 2C), and quantification of volumes showed that the PGZ was significantly smaller in cave animals than that of surface fish (Pachón = 10.4% decrease in volume compared to surface fish, Tinaja = 50.0% decrease in volume compared to surface fish, Molino = 31.3% decrease in volume compared to surface fish) (Figure 2E). These findings extend previous observations in Pachón cavefish to Molino and Tinaja (Soares et al., 2004), revealing convergence on reduced size of the optic tectum in adult cavefish populations.

### Expansion of the Telencephalon in Cavefish Populations

**FIGURE 3:**
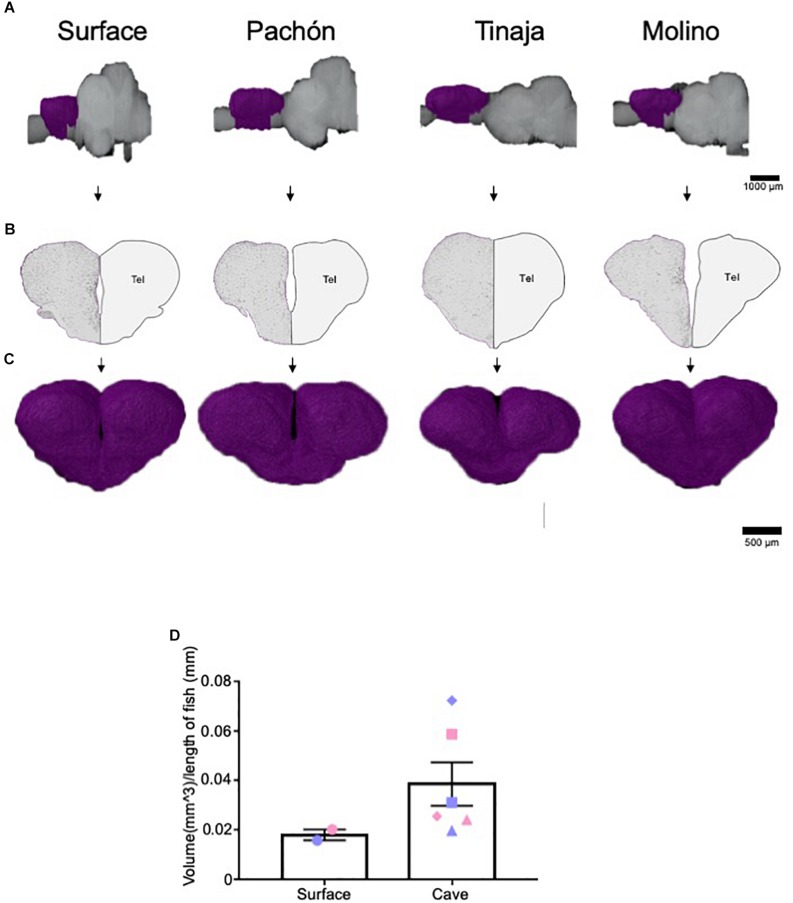
Expansion of telencephalon in cavefish populations. **(A)** 3-D reconstructions of Surface, Pachón, Tinaja, and Molino with demarcated telencephalon (purple) displayed. **(B)** Images of demarcated sections that were Nissl stained (left) and a cartoon of demarcated region (right). **(C)** Close up view of the 3-D reconstruction of the telencephalon from an anterior view. **(D)** The telencephalon from most cave animals was larger than that of surface conspecifics (Surface fish 0.018 ± 0.0022, Cave fish 0.039 ± 0.0088 *t*-test *t* = 1.276, df = 6, *p* = 0.12). Graph in panel **(D)** is the mean ± standard error of the mean. Blue points on bar graphs denote males, whereas light red denotes female. Square points on graphs represent Pachón, triangle points on graphs represent Tinaja and diamond points on graphs represent Molino.

The telencephalon modulates diverse behaviors that differ between surface and cavefish, including sleep, stress, and aggression (Kaslin et al., 2004; Portavella et al., 2004a, b; Elipot et al., 2013; Lal et al., 2018). Moreover, previous studies have mapped both the afferent and efferent projections of the telencephalon, as well as individual nuclei in the *A. mexicanus* brain (Riedel, 1997; Riedel and Krug, 1997). Given the importance of the telencephalon, we quantified telencephalic volume across *A. mexicanus* populations and found it to be expanded in all three populations of cavefish compared to surface fish (Figures 3A–C). Comparing total volume for surface fish and the combined data for cavefish populations revealed an increase in volume for all three cavefish populations, thought statistical significance was not reached (Figure 3D; Pachón = 150% increase in volume compared to surface fish, Tinaja = 22% increase in volume compared to surface fish, Molino = 172% increase in volume compared to surface fish). In addition, we observed differences in telencephalon shape between surface and cavefish populations. In all three cavefish populations the telencephalon is longer along the anterior-posterior axis than in surface fish (Figure 3A). Collectively, these data reveal a robust expansion of the telencephalon across three independent cavefish populations.

### Analysis of Thalamic and Habenular Nuclei

**FIGURE 4:**
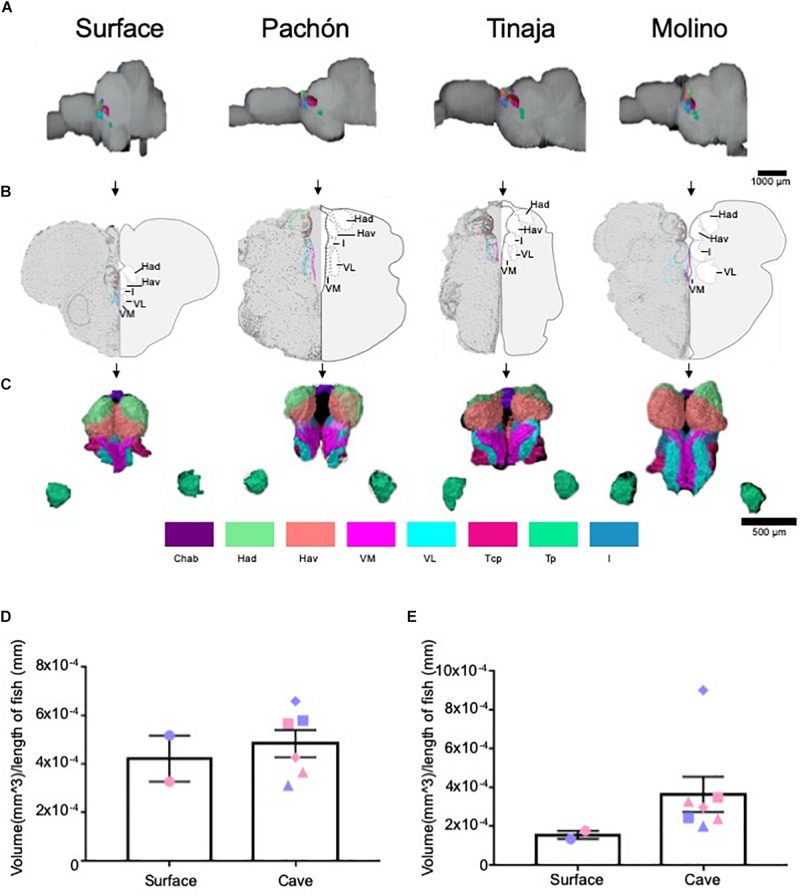
Quantification of the thalamus and habenulae reveals differences in some, but not all, subnuclei. **(A)** 3-D reconstructions of Surface, Pachón, Tinaja, and Molino with demarcated thalamus and habenula displayed. **(B)** Images of demarcated sections that were Nissl stained (left) and a cartoon of demarcated region (right). **(C)** 3-D reconstruction of subnuclei of the thalamus and habenulae, displayed from an anterior view. **(D)** Quantification of the total volume of the thalamus revealed no significant differences (surface fish 0.0004 ± 0.0001, cavefish 0.005 ± 0.0001, *t*-test *t* = 0.5519, df = 6, *p* = 0.30). **(E)** Quantification of the total volume of the habenulae revealed no significant differences (surface fish 0.00015 ± 0.00002, cavefish 0.0003 ± 0.00009, *t*-test *t* = 1.161, df = 6, *p* = 0.14). Graphs in panel **(D,E)** are the mean ± standard error of the mean. Color key for subnuclei (Chab = Habenula Commissure, Had = Dorsal Habenular Nucleus, Hav = Ventral Habenular Nucleus, VM = Ventromedial Prethalamus, VL = Ventrolateral Prethalamus, Tcp = Central posterior thalamic nucleus, Tp = Posterior thalamic nucleus, I = Intermediate Prethalamus). Blue points on bar graphs denote males, whereas light red denotes female. Square points on graphs represent Pachón, triangle points on graph represent Tinaja and diamond points on graph represent Molino.

The thalamus is a central relay unit connecting the forebrain with downstream mid- and hindbrain targets, and different regions of the thalamus have been shown in mammals to modulate diverse behaviors including stress, aggression, and sleep (Chauveau et al., 2009; Shin and Liberzon, 2010; Kim et al., 2012; Li et al., 2014; Latchoumane et al., 2017; Fernandez et al., 2018). Moreover, anatomy and function of thalamic nuclei are conserved among mammals and fish (Mueller and Wullimann, 2009; Amo et al., 2010; Chou et al., 2016; Duboué et al., 2017). Quantification of the entire thalamus revealed no significant differences in gross volume between cave and surface fish (Figures 4A–D; Pachón = 28.3% increase in volume compared to surface fish, Tinaja = 20.0% decrease in volume compared to surface fish, Molino = 28.3% increase in volume compared to surface fish). We then examined volumetric differences between thalamic subnuclei, including the posterior (Tp), anterior (Ta), and central posterior (Tcp), as well as the lateral (VL), medial (VM) and intermediate (I) prethalamus [formerly called ventrolateral, ventromedial thalamus (Mueller, 2012)] (Supplementary Figure S1). Of these, the posterior thalamic nucleus and the medial prethalamus were significantly larger in the cavefish populations (Supplementary Figure S1). By contrast, no differences were observed for the other thalamic and prethalamic nuclei we examined (Supplementary Figures S1C–F); however, while not significant, all volumetric measurements for the anterior thalamic nucleus from cavefish were larger than those of the surface fish we scored (Supplementary Figure S1E).

The habenular nuclei are a conserved brain regions that also connect forebrain to midbrain (Sutherland, 1982; Viswanath et al., 2014). In rodents and other mammals, the habenulae have been shown to regulate diverse behaviors, including sleep, stress, feeding, and social interactions (Murray et al., 1994, Murphy et al., 1996; van Kerkhof et al., 2013; Stamatakis et al., 2016; Haun et al., 2018). Recently, the habenular nuclei have also been found to modulate similar behaviors in zebrafish (Agetsuma et al., 2010; Chou et al., 2016; Duboué et al., 2017). Because many of the behaviors modulated by the habenulae differ between surface fish and cavefish populations, we examined volumes of individual nuclei within the habenulae. The habenulae are comprised of the dorsal and ventral habenula, and its commissure (Duboué and Halpern, 2017), and this neuroanatomy is conserved among vertebrates (deCarvalho et al., 2014). The entire habenula was enlarged in all cavefish populations (Figures 4A–C,E; Pachón = 89.2% increase in volume compared to surface fish, Tinaja = 63.1% increase in volume compared to surface fish, Molino = 282.2% increase in volume compared to surface fish). Examining individual nuclei revealed an expansion of ventral habenular nucleus (Hav) in all cavefish examined, relative to surface fish though this did not reach statistical significance (Supplementary Figure S2A). By contrast, no differences were found in volumes of the dorsal habenulae (Had) or in the habenular commissure (Chab) (Supplementary Figures S2B,C). Taken together, these findings reveal differences within the ventral habenula of all cavefish studies relative to surface animals.

### Analysis of the Hypothalamus Reveal Evolutionary Changes to Some but Not All Subnuclei

**FIGURE 5:**
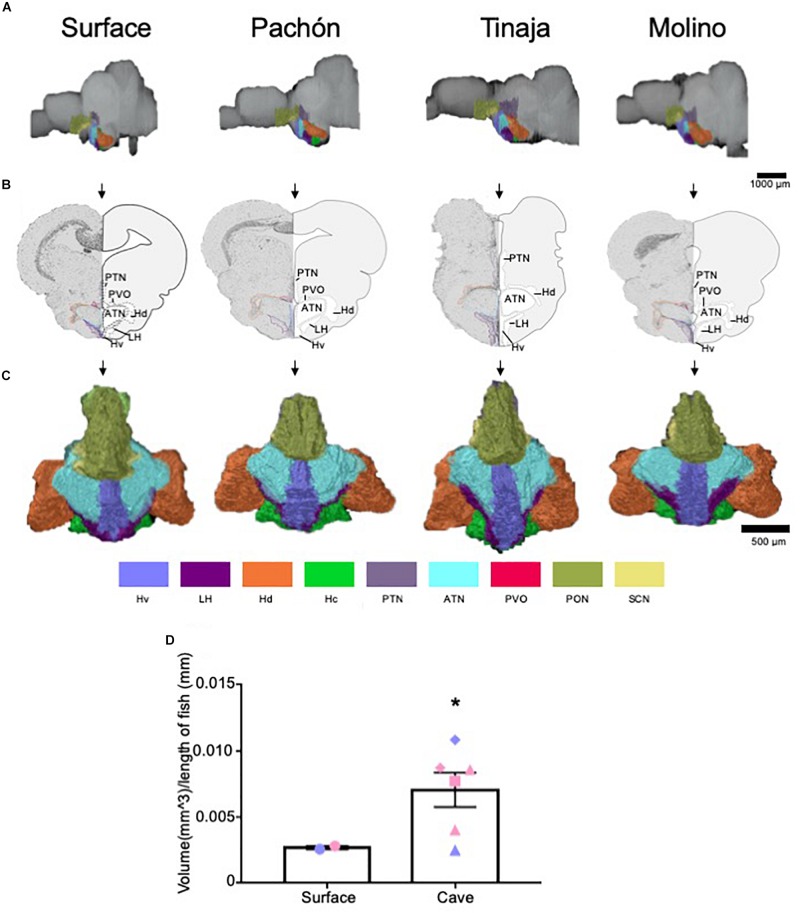
Quantification of the hypothalamus reveals expansion in cave populations. **(A)** 3-D reconstructions of Surface, Pachón, Tinaja and Molino with demarcated hypothalamus displayed. **(B)** Images of demarcated sections that were Nissl stained (left) and a cartoon of demarcated region (right). **(C)** 3-D reconstruction of the hypothalamus only, displayed from an anterior view. **(D)** Quantification of the total volume of the hypothalamus revealed expansion in cavefish (surface fish 0.0027 ± 0.0001, cavefish 0.0071 ± 0.0013, *t*-test *t* = 1.855, df = 6, *p* ≤ 0.05). Graph in **D** is the mean ± standard error of the mean. Color key for subnuclei (Hv = Ventral zone of periventricular hypothalamus, LH = Lateral hypothalamic nucleus, Hd = Dorsal zone of periventricular hypothalamus, Hc = Caudal zone of periventricular hypothalamus, PTN = Posterior tuberculum, ATN = Anterior tuberculum, PVO = Paraventricular organ PON = Preoptic nucleus, SCN = Superchiasmatic nucleus). Blue points on bar graphs denote males, whereas light red denotes female. Square points on graphs represent Pachón, triangle points on graphs represent Tinaja and diamond points on graphs represent Molino.

The hypothalamus controls numerous homeostatically regulated behaviors that are known to differ between surface fish and cavefish, including sleep, feeding, stress, and social behaviors (Duboué et al., 2011; Kowalko et al., 2013a, b; Chin et al., 2018; Lloyd et al., 2018; Zha and Xu, 2015). To determine whether these behavioral changes are accompanied by alterations in anatomy, we quantified the overall size of the hypothalamus, as well as individual subnuclei that modulate distinct behaviors in mammals (Figure 5 and Supplementary Figure S3). We found the total volume of the hypothalamus was significantly enlarged in the cavefish populations compared to surface fish (Figures 5A–D and Supplementary Movies S5–S8; Pachón = 185.2% increase in volume compared to surface fish, Tinaja = 85% increase in volume compared to surface fish, Molino = 263% increase in volume compared to surface fish). An expanded hypothalamus in cavefish has been demonstrated previously for larval forms (Menuet et al., 2007), and thus these data reveal that hypothalamic expansion is conserved through adulthood.

We next examined the volume of different hypothalamic subnuclei (Supplementary Figure S3). We first measured the suprachiasmatic nucleus (SCN). The SCN is a critical regulator of circadian rhythms in mammals (Moore, 1982; Moore and Whitmore, 2014). Surprisingly, no significant differences were observed in the SCN between surface and cavefish animals (Supplementary Figure S3A). By contrast, the size of the lateral hypothalamic nucleus, the ventral zone of periventricular hypothalamus, the paraventricular organ, caudal zone of periventricular hypothalamus, preoptic nucleus, the anterior tuberculum, and the posterior tuberculum appeared qualitatively larger in most cave individuals we examined. When we pooled the volumes of the cavefish and performed statistical analysis, we found the lateral hypothalamic nucleus, ventral zone of periventricular hypothalamus and the paraventricular organ were enlarged significantly in cave animals (Supplementary Figures S3B–D). Moreover, the volumes of the caudal zone of the periventricular hypothalamus and posterior tuberculum were found to approach significance (Supplementary Figures S3E,F, *p* = 0.07 for both). By contrast, whereas the volumes for the other hypothalamic nuclei were larger in most cave animals, the values did not reach statistical significance (Supplementary Figures S3E–I).

## Discussion

We have generated an adult brain atlas for surface fish and three cavefish populations of *A. mexicanus*. A detailed adult brain atlas has been previously generated in zebrafish (Wulliman et al., 1996), and another brain atlas has been published in a cave/surface hybrid population of *A. mexicanus* cavefish (though it is untranslated from German) (Natke, 1999). These two resources provide a point of comparison for identifying neuroanatomical loci in cave and surface populations of *A. mexicanus*. An estimated ∼100–250 million years ago of divergence separate *A. mexicanus* and *Danio rerio* (Peng et al., 2006; Nakatani et al., 2011). We found that the gross neuroanatomy of *A. mexicanus* and zebrafish were largely similar, allowing for identification major brain structures.

Our analysis provides the first comparative brain atlas for surface and cave populations of *A. mexicanus*. The use of automated serial sectioning allows for volumetric reconstruction of brain regions and semi-quantitative comparisons of neuroanatomy between surface and cavefish populations. While this approach is technically feasible, practically it is limited due to the labor-intensive nature of manually tracing brain regions, and difficulties obtaining completely sectioned brains. In this study, we chose to focus on the visual system as a proof-of-principle, as well as the hypothalamus, thalamus, and habenula due to their known role in behavioral regulation. While the small number of replicates largely prevented statistical comparisons between individual cavefish populations, the robust volume differences observed between surface and cave populations for many brain regions suggest this approach may be practical for detailed anatomical comparison. Here, we have made all raw data available so that others may quantify additional brain regions of interest (Tables 1, 2, and data available upon request).

Brain atlases have been widely used in a number of species, including zebrafish, and have expanded greatly our understanding of how individual neuronal areas modulate myriad behaviors (Wulliman et al., 1996; Hawrylycz et al., 2011; Peng et al., 2011; Marquart et al., 2015; Milyaev et al., 2012; Mueller and Wullimann, 2015; Randlett et al., 2015). Brain atlases have been generated in larval zebrafish that provide near single-neuron resolution of brain structures (Ronneberger et al., 2012; Marquart et al., 2015; Randlett et al., 2015; Dunn et al., 2016). The transparency of zebrafish larvae allows for the application of functional imaging approaches (Ahrens et al., 2013; Muto et al., 2013), that can then be mapped on brain atlases to identify changes in activity within defined neurons (Dunn et al., 2016). *A. mexicanus* larvae, like zebrafish, are transparent, providing potential for the generation of a high-resolution brain atlas.

While the level of accuracy obtained with a larval atlas is not possible in adult fish using currently available technology due to the larger size of the brain and the need for sectioning, the added complexity of the adult brain and its similarity to rodents is particularly effective in comparative neuroanatomy. Further, a number of behaviors that differ between surface and cave individuals are not present in larval forms. For example, a loss of aggressive behavior has been documented in cavefish animals (Elipot et al., 2013), and other studies have demonstrated that cavefish do not school, whereas their surface conspecifics do (Kowalko et al., 2013b). Many behaviors, such as vibration attraction behavior, schooling, and differences in aggression, are not present in larval forms (Yoshizawa et al., 2010; Elipot et al., 2013; Kowalko et al., 2013b), and thus an adult atlas facilitates identification of brain regions that modulate more complex behaviors only seen in adults.

In this study, brain regions were standardized to the length of each individual fish, from the anterior most region of the nose to the beginning of the caudal tail. To correct for individual differences in size and growth rate, we normalized all brain volumes (Gallo and Jeffery, 2012). Quantitative comparisons between smaller neuroanatomical regions, such as individual nuclei within the hypothalamus or thalamus, may be confounded by large differences within other brain regions, such as the optic tectum. However, the variability in differences between subnuclei suggests localized changes in brain volume can be detected. As an example, most nuclei in the hypothalamus are expanded across cavefish populations, yet no differences are detected within the SCN for cavefish relative to surface.

Our findings identify the expansion of multiple hypothalamic nuclei, suggesting shared processes may govern evolved differences in hypothalamic development. The hypothalamus in cavefish larvae is expanded through a mechanism that is dependent on the differential expression of several morphogens and transcription factors, including sonic hedgehog and Nkx2.1 (Menuet et al., 2007). One hypothesis is that reduced anatomical constraints from eye-loss allow for hypothalamic expansion. A number of hypothalamic neuropeptides are known to be upregulated in cavefish including HCRT and NPY, which localize to the lateral hypothalamus and periventricular/lateral hypothalamus respectively (Alié et al., 2018; Jaggard et al., 2018; Jeong et al., 2018). Both of these nuclei are larger across all three populations of cavefish. Many hypothalamus-regulated behaviors including sleep (PON), feeding (Hl, PVN), aggression (PON), and sociality (PON) are altered in cavefish (Duboué et al., 2011; Elipot et al., 2013; Kowalko et al., 2013a, b; Miyasaka et al., 2014; Jaggard et al., 2018; Lloyd et al., 2018), suggesting hypothalamic function may be a under significant selective pressure.

In agreement with the previous literature, we identified convergent evolution of changes in brain regions associated with sensory processing (Soares et al., 2004; Moran et al., 2015; Hinaux et al., 2016). The optic tectum is significantly reduced across all three cavefish populations. These findings are consistent with an increased reliance on non-visual cues in cave animals (Bradic et al., 2013; Yoshizawa et al., 2013). This atlas allows for future studies examining the neuroanatomy of brain regions associated with non-visual cues. For example, taste buds are more numerous in cavefish (Bibliowicz et al., 2013; Hinaux et al., 2016) and the lateral line of cavefish is also significantly expanded, suggesting increased reliance on sensory processes that do not involve sight (Teyke, 1990; Yoshizawa et al., 2014). The sensory neurons from taste and mechanosensation neurons project to the nucleus of the solitary tract (NST) and medial octavolateralis nucleus (MON) within the brain, respectively (Puzdrowski, 1989; McCormick and Braford, 1994; Vendrell-Llopis and Yaksi, 2015). Based on findings from other sensory pathways, these regions may be predicted to be enlarged. Future analysis of serially sectioned brains will allow for detailed quantification and comparison of sensory structures between *A. mexicanus* populations.

Here, we used brains stained with Nissl, and demarcated manually individual regions of the adult brain. We see two main future expansions of this work. First, future efforts will streamline the labor-intensive approach of manual demarcation of individual regions. Similar large-scale neuroanatomical reconstruction efforts, such as electron microscopy tracing of the *Drosophila* brain have been successful in analyzing large data sets like these (Zheng et al., 2018). It is also possible that automated tracing methodology may be developed to reduce the time required for analysis. Further, future imaging of additional serially-section brains may allow for more quantitative comparisons between populations. Second, in zebrafish and other models, transgenic labeling of precise neuronal population has facilitated greatly the demarcation of individual neuronal regions (Kawakami et al., 2000; Scott et al., 2007). Moreover, transgenic labeling of neurons in the brain permits tracing of neuronal projections, something that is not possible with Nissl staining (deCarvalho et al., 2013; Miyasaka et al., 2014). Whereas transgenic technology has not been widely used in *A. mexicanus*, recent studies have shown that the Tol2 system, which is widely used in zebrafish, is highly effective in *A. mexicanus* surface and cavefish (Elipot et al., 2014; Stahl et al., 2019a, b). Future work incorporating these tools would facilitate a highly defined neuroanatomical brain atlas for the *A. mexicanus* adult brain.

## Materials and Methods

### Fish Husbandry

Animal care and husbandry was carried out as previously described (Borowsky, 2008; Jaggard et al., 2018). Briefly, adult *A. mexicanus* stocks were originally obtained from the Jeffery (University of Maryland) or Borowsky laboratories (New York University). These fish have been bred and maintained on a recirculating aquatics system at Florida Atlantic University. The water temperature was maintained at 23 ± 1°C, and the lights were maintained on a 14:10 LD cycle (25–40 lux at lights on). All fish were fed a mix of fish flakes (TetraMin) and California black worms (Aquatic Foods). All experiments in this study were approved by the Institutional Animal Care and Usage Committee (IACUC) at Florida Atlantic University, protocol numbers A17–21 and A15–32, or the IACUC at Stowers Institute for Medical Research. All fish used in this study were approximately 1 year old. A total of 10 brains were dissected and analyzed per population. We used 1 male and 1 female brains from surface population, 1 male and 1 female brains from Pachón population, 1 male and 2 female brains from Tinaja population, and 1 male and 1 female brains from Molino population. In some cases, brains could not be quantified for all neuroanatomical regions due to tissue damage.

### Sectioning

Fish were euthanized by incubation in MS-222 (500 mg/L) for 10 min and decapitated using sharp scissors. The head was immediately fixed with freshly prepared 4% paraformaldehyde (PFA, diluted from 16% (wt/vol) aqueous solution, Electron Microscopy Sciences, cat# 15710) in 1 × PBS for 48 h at 4°C with a change of 4% PFA/1×PBS after 3 h. Heads were washed three times in 1×PBS and subsequently, brains were dissected according to Moran et al. (2015). Brains were dehydrated through graded ethanol (30, 50, and 70%) and processed with a PATHOS Delta hybrid tissue processor (Milestone Medical Technologies, Inc., MI) followed by paraffin embedding. Coronal slices of paraffin sections with 8 μm thickness were continuously cut using a Leica RM2255 microtome (Leica Biosystems Inc., Buffalo Grove, IL, United States) and mounted on Superfrost Plus microscope slides (cat# 12-550-15, Thermo Fisher Scientific). Nissl staining was performed as described in Vacca (1985). Briefly, sections were deparaffinized and hydrated in distilled water. Sections were stained in cresyl echt violet (0.5 g cresyl echt violet (CI 51010); 80 mL distilled water; 20 mL absolute alcohol) for 8 min, briefly rinsed in distilled water, dehydrated with 95% absolute alcohol 2 times, subsequently cleared in 2 changes of xylene and finally mounted. Slides were scanned using an Olympus slide scanner VS120 with a 20× objective. Images were extracted from VSI files in sequence using a customized plugin in Fiji (ver 1.51H) (Schindelin et al., 2012), a mask constructed, and registered using a multithreaded version of StackReg1 (Thévenaz et al., 1998). Blank spaces in the registered image were filled with artificial noise that matched the all-white background using a custom plugin in Fiji. Plugins are available at https://github.com/jouyun/smc-plugins and https://github.com/cwood1967/IJPlugins/.

### Volumetric Reconstruction

ImageJ FIJI (ver 1.51H) (Schindelin et al., 2012) was used to convert serial sections to a .tif image sequence. Image sequence was uploaded into the AMIRA software (ver 6.2.0, Thermo Fisher, Waltham, MA, United States). To create proper demarcations, neuroanatomical regions of interest (ROIs) from Nissl stains were set under the “segmentation” tab using the lasso tool. To view 3-dimensional reconstructions of neuroanatomical ROI’s, a “volren” object was created under the “project” tab. Each volren object was connected to the original .tif image sequence as well as the label fields used to create demarcated neuroanatomical ROI’s.

### Measurements and Statistical Analysis

To quantify total volume of induvial demarcated regions (i.e., each ROI), we used the “volume per VOI” result of the “material statistics” function in AMIRA (ver 6.2.0). To correct for differences in size and growth rate among different fish populations, all volumetric results were normalized to the length of the fish, from the anterior nose to the caudal tail. Volumetric measurements were thus calculated as a ratio of volume relative to this length. For statistical comparisons of ROI volumes between two groups (i.e., the pooled cavefish data compared to surface), we used a standard *t*-test. All statistics were performed using GraphPad Prism (ver 7.0).

## Data Availability Statement

Original data underlying this manuscript can be accessed from the Stowers Original Data Repository at http://www.stowers.org/research/publications/libpb-1427. All original and analyzed data will also be provided upon request.

## Ethics Statement

The animal study was reviewed and approved by Florida Atlantic University Institutional Animal Care and Usage Committee, protocol numbers A17-21 and A15-32 Florida Atlantic University Institutional Animal Care and Usage Committee, protocol number 2019-084 Stowers Institutional Animal Care and Usage Committee.

## Author Contributions

CL, NR, SEM, AK, and ED designed the experiments. RP, YW, SAM, and NR collected brains and performed serial sectioning, and registration. CL oversaw analysis of data. CL, JJ, AR, SR, DW, and MG analyzed the data. CL, AK, and ED wrote the manuscript with significant input from SAM, NR, and RP.

## Conflict of Interest

The authors declare that the research was conducted in the absence of any commercial or financial relationships that could be construed as a potential conflict of interest.

## Supplementary Material

FIGURE S1 Analysis of different thalamic subnuclei reveals expansion of some, but not all, regions. **(A)** Quantification of the posterior thalamic nucleus shows a significant expansion of volume in cavefish (surface fish 3.07e-5 ± 5.2e-6, cavefish 5.35e-5 ± 2.34e-6, *t*-test *t* = 4.637, df, *p* < 0.05). **(B)** Analysis of medial prethalamus shows a significant expansion in volume of cave fish (surface fish 2.96e-5 ± 8.6e-6, cavefish 5.16e-5 ± 4.23e-6, *t*-test *t* = 2.524, df = 6, *p* < 0.05). **(C–F)** There were no differences between the lateral prethalamus **(C)**, intermediate prethalamus **(D)**, anterior thalamic nucleus **(E)** and central posterior thalamic nucleus **(F)** (VL = surface fish 9.04e-5 ± 4.86e-5, cavefish 0.0001 ± 1.81e-5, *t*-test *t* = 0.298, df = 6, *p* = 0.38; I = surface fish 1.47e-5 ± 6.34e-6, cavefish 1.55e-5 ± 2.05e-6, *t*-test *t* = 1.823, df = 6, *p* = 0.43; Ta = surface fish 1.45e-5 ± 2.5e-7, cavefish 6.93e-5 ± 1.72e-5, *t*-test *t* = 1.751, df = 6, *p* = 0.06; Tcp = surface fish 0.0002 ± 0.0001, cavefish 0.0001 ± 2.83e-5, *t*-test *t* = 0.8033, df = 6, *p* = 0.22). All graphs are the mean ± standard error of the mean. Blue points on bar graphs denote males, whereas light red denotes female. Asterisk represent significance below *p* = 0.05. Square points on graphs represent Pachón, triangle points on graphs represent Tinaja and diamond points on graphs represent Molino.

FIGURE S2 Analysis of different habenulae subnuclei reveals expansion of subnuclei, some approaching significance. **(A)** Quantification of the ventral habenular nucleus shows a general expansion in cavefish (surface fish 8.12e-5 = 3.5e-6, cavefish 0.0002 = 4.45e-5, *t*-test t = 1.206, df = 7, *p* = 0.13). **(B)** Quantification of dorsal habenular nucleus shows no difference between surface and cavefish (surface fish 0.0002 ± 0.0001, cavefish 0.0002 ± 4.48e-5, *t*-test *t* = 0.198, df = 7, *p* = 0.42). **(C)** Analysis of the habenula commissure showed no significance between morphs (surface fish 5.67e-6 ± 1.57e-6, cavefish 1.05e-5 ± 4.94e-6, *t*-test *t* = 0.4997, df = 7, *p* = 0.31). All graphs are the mean ± standard error of the mean. Blue points on bar graphs denote males, whereas light red denotes female. Square points on graphs represent Pachón, triangle points on graphs represent Tinaja and diamond points on graphs represent Molino.

FIGURE S3 Analysis of different hypothalamic subnuclei reveals significant expansion of lateral, dorsal, and caudal hypothalamus in cavefish while others remain similar to surface fish. **(A)** Analysis of the suprachiasmatic nucleus showed no difference between morphs (surface fish 5.69e-5 ± 5.01e-5, cavefish 0.0001 ± 2.92e-5, *t*-test *t* = 1.369, df = 6, *p* = 0.11). **(B–D)** Significant differences were observed between the lateral hypothalamic nucleus (LH) **(B)**, ventral zone of periventricular hypothalamus (Hv) **(C)** and paraventricular organ (PVO) **(D)** (LH = surface fish 0.0001 ± 2.55e-5, cavefish 0.0003 ± 4.46e-5, *t*-test *t* = 2.062, df = 7, *p* < 0.05; Hv = surface fish 0.0002 ± 5e-6, cavefish 0.0004 ± 6.37e-5, *t*-test *t* = 2.01, df = 7, *p* < 0.05; PVO = surface fish 3.59e-5 ± 1.16e-5, cavefish 9.79e-5 ± 1.47e-5, *t*-test *t* = 2.242, df = 6, *p* < 0.05). **(E,F)** Analysis of the caudal zone of periventricular hypothalamus (Hc) **(E)**, posterior tuberculum (PTN) **(F)** showed an enlargement in cavefish that approached significance (Hc = surface fish 0.0002 ± 1.58e-5, cavefish 0.0008 ± 0.0002, *t*-test *t* = 1.603, df = 7, *p* = 0.07; PTN = surface fish 0.001 ± 2.85e-5, cavefish 0.0004 ± 8.71e-5, *t*-test *t* = 1.627, df = 6, *p* = 0.07). **(G–I)** Quantification of the anterior tuberculum (ATN) **(G)**, dorsal zone of periventricular hypothalamus (Hd) **(H)** and preoptic nucleus (PON) (I) show no difference between surface and cavefish (ATN = surface fish 0.0007 ± 7.5e-5, cavefish 0.0013 ± 0.0003 *t*-test *t* = 1.283, df = 6, *p* = 0.12; Hd = Surface fish 0.0006 ± 7.85e-5, Cave fish 0.0015 ± 0.0003, *t*-test *t* = 1.345, df = 7, *p* = 0.11; PON = Surface fish 0.0005 ± 4.95e-5, Cave fish 0.0011 ± 0.0002, *t*-test *t* = 1.275, df = 6, *p* = 0.12). All graphs are the mean ± standard error of the mean. Asterisk represent significance below *p* = 0.05. Blue points on bar graphs denote males, whereas light red denotes female. Square points on graphs represent Pachón, triangle points on graphs represent Tinaja and diamond points on graphs represent Molino.

MOVIE S1 Three-dimensional reconstruction of whole brain from surface fish.

MOVIE S2 Three-dimensional reconstruction of whole brain from Pachón cavefish.

MOVIE S3 Three-dimensional reconstruction of whole brain from Tinaja cavefish.

MOVIE S4 Three-dimensional reconstruction of whole brain from Molino cavefish.

MOVIE S5 Three-dimensional reconstruction of hypothalamus from surface cavefish.

MOVIE S6 Three-dimensional reconstruction of hypothalamus from Pachón cavefish.

MOVIE S7 Three-dimensional reconstruction of hypothalamus from Tinaja cavefish.

MOVIE S8 Three-dimensional reconstruction of hypothalamus from Molino cavefish.
