# Supplementary figures and images for: An Adult Brain Atlas Reveals Broad Neuroanatomical Changes in Independently Evolved Populations of Mexican Cavefish
Source: Front Neuroanat. 2019 Oct 4;13:88. doi: 10.3389/fnana.2019.00088 (PMC6788135; doi:10.3389/fnana.2019.00088)

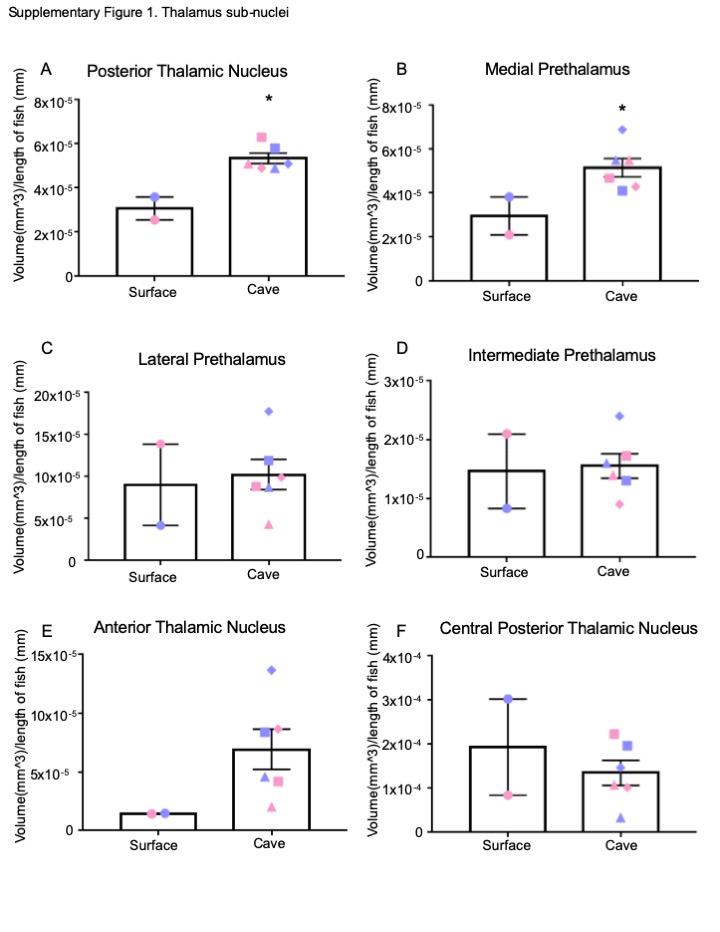

Supplement: FIGURE S1 — Analysis of different thalamic subnuclei reveals expansion of some, but not all, regions. (A) Quantification of the posterior thalamic nucleus shows a significant expansion of volume in cavefish (surface fish 3.07e-5 ± 5.2e-6, cavefish 5.35e-5 ± 2.34e-6, t-test t = 4.637, df, p < 0.05). (B) Analysis of medial prethalamus shows a significant expansion in volume of cave fish (surface fish 2.96e-5 ± 8.6e-6, cavefish 5.16e-5 ± 4.23e-6, t-test t = 2.524, df = 6, p < 0.05). (C–F) There were no differences between the lateral prethalamus (C), intermediate prethalamus (D), anterior thalamic nucleus (E) and central posterior thalamic nucleus (F) (VL = surface fish 9.04e-5 ± 4.86e-5, cavefish 0.0001 ± 1.81e-5, t-test t = 0.298, df = 6, p = 0.38; I = surface fish 1.47e-5 ± 6.34e-6, cavefish 1.55e-5 ± 2.05e-6, t-test t = 1.823, df = 6, p = 0.43; Ta = surface fish 1.45e-5 ± 2.5e-7, cavefish 6.93e-5 ± 1.72e-5, t-test t = 1.751, df = 6, p = 0.06; Tcp = surface fish 0.0002 ± 0.0001, cavefish 0.0001 ± 2.83e-5, t-test t = 0.8033, df = 6, p = 0.22). All graphs are the mean ± standard error of the mean. Blue points on bar graphs denote males, whereas light red denotes female. Asterisk represent significance below p = 0.05. Square points on graphs represent Pachón, triangle points on graphs represent Tinaja and diamond points on graphs represent Molino. [file Image_1.JPEG]

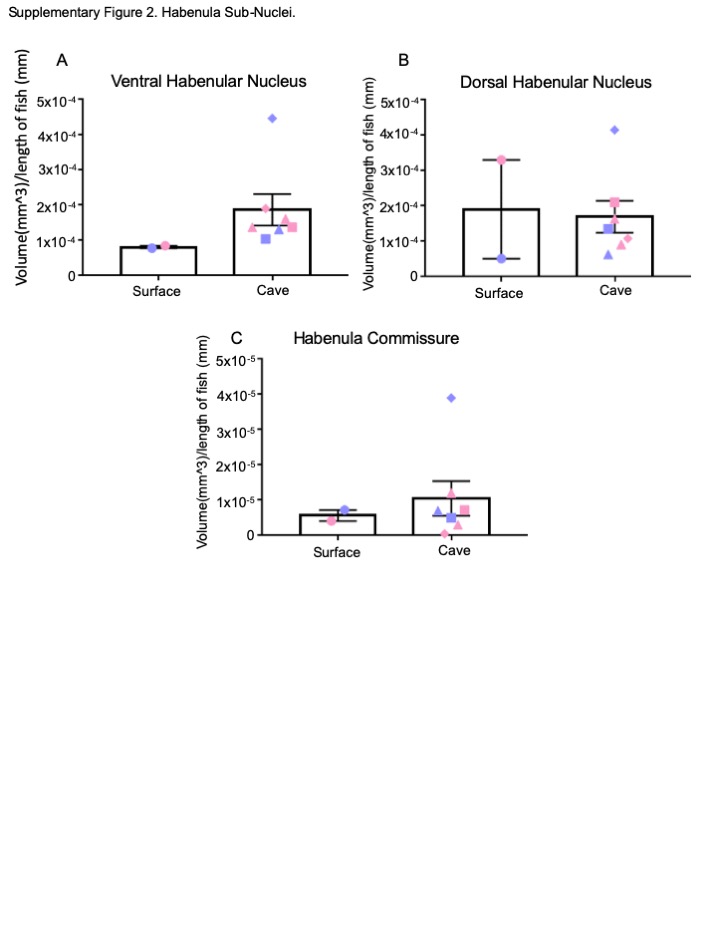

Supplement: FIGURE S2 — Analysis of different habenulae subnuclei reveals expansion of subnuclei, some approaching significance. (A) Quantification of the ventral habenular nucleus shows a general expansion in cavefish (surface fish 8.12e-5 = 3.5e-6, cavefish 0.0002 = 4.45e-5, t-test t = 1.206, df = 7, p = 0.13). (B) Quantification of dorsal habenular nucleus shows no difference between surface and cavefish (surface fish 0.0002 ± 0.0001, cavefish 0.0002 ± 4.48e-5, t-test t = 0.198, df = 7, p = 0.42). (C) Analysis of the habenula commissure showed no significance between morphs (surface fish 5.67e-6 ± 1.57e-6, cavefish 1.05e-5 ± 4.94e-6, t-test t = 0.4997, df = 7, p = 0.31). All graphs are the mean ± standard error of the mean. Blue points on bar graphs denote males, whereas light red denotes female. Square points on graphs represent Pachón, triangle points on graphs represent Tinaja and diamond points on graphs represent Molino. [file Image_2.JPEG]

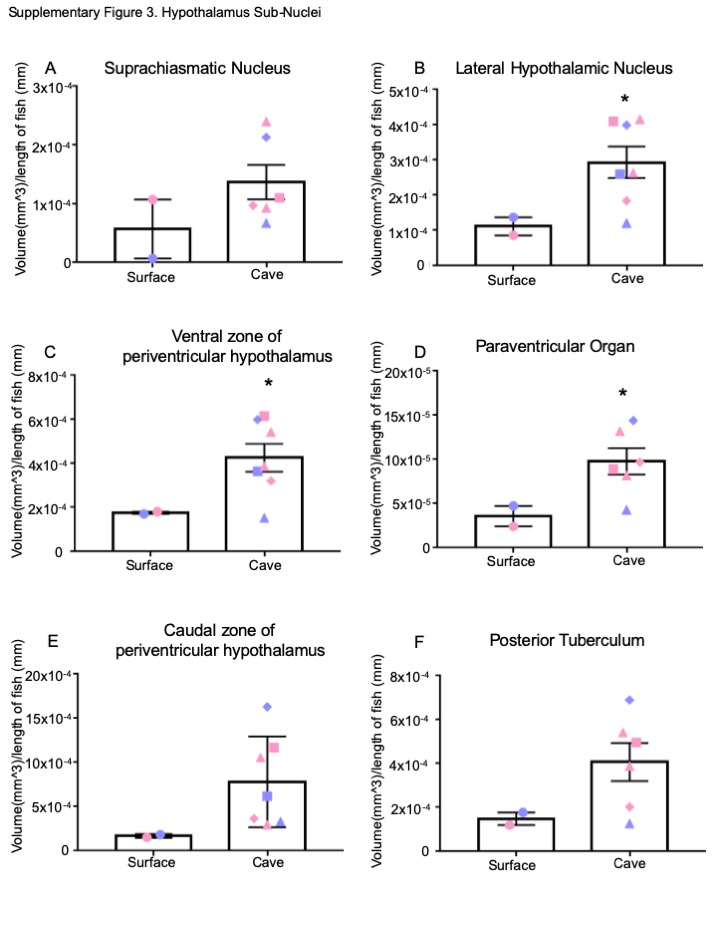

Supplement: FIGURE S3 — Analysis of different hypothalamic subnuclei reveals significant expansion of lateral, dorsal, and caudal hypothalamus in cavefish while others remain similar to surface fish. (A) Analysis of the suprachiasmatic nucleus showed no difference between morphs (surface fish 5.69e-5 ± 5.01e-5, cavefish 0.0001 ± 2.92e-5, t-test t = 1.369, df = 6, p = 0.11). (B–D) Significant differences were observed between the lateral hypothalamic nucleus (LH) (B), ventral zone of periventricular hypothalamus (Hv) (C) and paraventricular organ (PVO) (D) (LH = surface fish 0.0001 ± 2.55e-5, cavefish 0.0003 ± 4.46e-5, t-test t = 2.062, df = 7, p < 0.05; Hv = surface fish 0.0002 ± 5e-6, cavefish 0.0004 ± 6.37e-5, t-test t = 2.01, df = 7, p < 0.05; PVO = surface fish 3.59e-5 ± 1.16e-5, cavefish 9.79e-5 ± 1.47e-5, t-test t = 2.242, df = 6, p < 0.05). (E,F) Analysis of the caudal zone of periventricular hypothalamus (Hc) (E), posterior tuberculum (PTN) (F) showed an enlargement in cavefish that approached significance (Hc = surface fish 0.0002 ± 1.58e-5, cavefish 0.0008 ± 0.0002, t-test t = 1.603, df = 7, p = 0.07; PTN = surface fish 0.001 ± 2.85e-5, cavefish 0.0004 ± 8.71e-5, t-test t = 1.627, df = 6, p = 0.07). (G–I) Quantification of the anterior tuberculum (ATN) (G), dorsal zone of periventricular hypothalamus (Hd) (H) and preoptic nucleus (PON) (I) show no difference between surface and cavefish (ATN = surface fish 0.0007 ± 7.5e-5, cavefish 0.0013 ± 0.0003 t-test t = 1.283, df = 6, p = 0.12; Hd = Surface fish 0.0006 ± 7.85e-5, Cave fish 0.0015 ± 0.0003, t-test t = 1.345, df = 7, p = 0.11; PON = Surface fish 0.0005 ± 4.95e-5, Cave fish 0.0011 ± 0.0002, t-test t = 1.275, df = 6, p = 0.12). All graphs are the mean ± standard error of the mean. Asterisk represent significance below p = 0.05. Blue points on bar graphs denote males, whereas light red denotes female. Square points on graphs represent Pachón, triangle points on graphs represent Tinaja and diamond points on graphs represent Molino [file Image_3.JPEG]

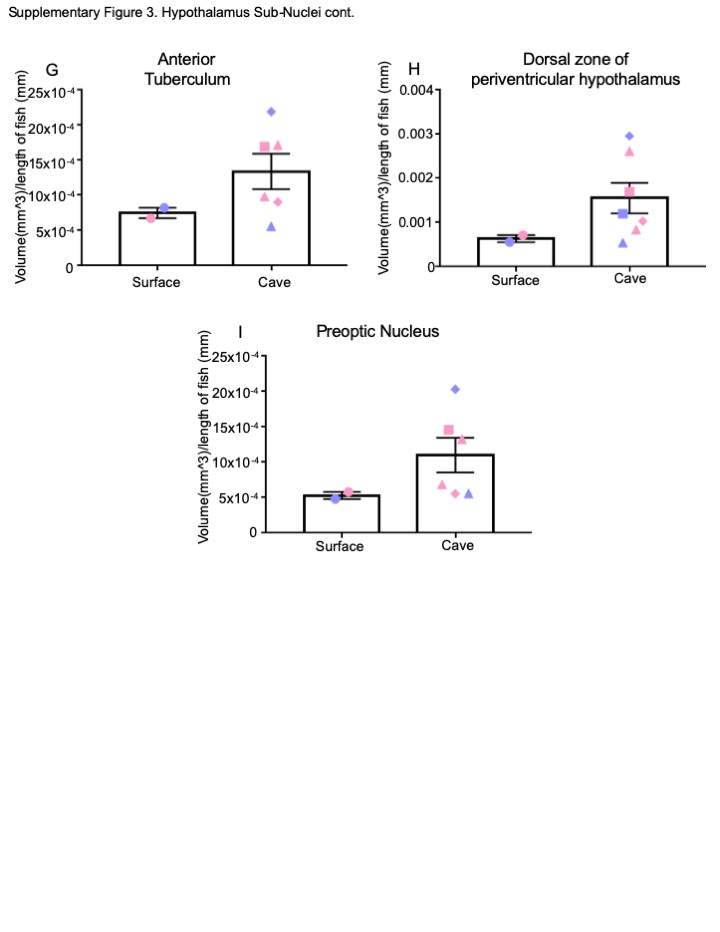

Supplement: Supplementary file 12 [file Image_4.JPEG]
